# Calcitonin gene-related peptide induces headache attacks in people with idiopathic intracranial hypertension

**DOI:** 10.1093/brain/awag126

**Published:** 2026-04-16

**Authors:** Andreas Yiangou, Thien Phu Do, Susan P Mollan, Mark Thaller, James L Mitchell, Caroline W Mugo, Georgios Tsermoulas, Lisa J Hill, Samuel J E Lucas, Messoud Ashina, Alexandra J Sinclair

**Affiliations:** Metabolism and Systems Science, College of Medicine and Health, University of Birmingham, Birmingham B15 2TT, UK; Department of Neurology, University Hospitals Birmingham NHS Foundation Trust, Birmingham B15 2GW, UK; Department of Neurology, Centre for Discoveries in Migraine, Danish Headache Centre, Copenhagen University Hospital-Rigshospitalet, Copenhagen 2600, Denmark; Metabolism and Systems Science, College of Medicine and Health, University of Birmingham, Birmingham B15 2TT, UK; Ophthalmology Department, Queen’s University, Kingston, Ontario K7L 4V7, Canada; Metabolism and Systems Science, College of Medicine and Health, University of Birmingham, Birmingham B15 2TT, UK; Department of Neurology, University Hospitals Birmingham NHS Foundation Trust, Birmingham B15 2GW, UK; Metabolism and Systems Science, College of Medicine and Health, University of Birmingham, Birmingham B15 2TT, UK; Department of Neurology, University Hospitals Birmingham NHS Foundation Trust, Birmingham B15 2GW, UK; Academic Department of Military Rehabilitation, Defence Medical Rehabilitation Centre, Stanford Hall LE12 5QW, UK; Metabolism and Systems Science, College of Medicine and Health, University of Birmingham, Birmingham B15 2TT, UK; School of Biomedical Sciences, Institute of Clinical Sciences, University of Birmingham, Birmingham B15 2TT, UK; Metabolism and Systems Science, College of Medicine and Health, University of Birmingham, Birmingham B15 2TT, UK; Department of Neurology, University Hospitals Birmingham NHS Foundation Trust, Birmingham B15 2GW, UK; School of Biomedical Sciences, Institute of Clinical Sciences, University of Birmingham, Birmingham B15 2TT, UK; School of Sport, Exercise and Rehabilitation Sciences, University of Birmingham, Birmingham B15 2TT, UK; Centre for Human Brain Health, University of Birmingham, Birmingham B15 2TT, UK; Department of Neurology, Centre for Discoveries in Migraine, Danish Headache Centre, Copenhagen University Hospital-Rigshospitalet, Copenhagen 2600, Denmark; Department of Clinical Medicine, University of Copenhagen, Copenhagen 2200, Denmark; Metabolism and Systems Science, College of Medicine and Health, University of Birmingham, Birmingham B15 2TT, UK; Department of Neurology, University Hospitals Birmingham NHS Foundation Trust, Birmingham B15 2GW, UK

**Keywords:** calcitonin gene-related peptide (CGRP), idiopathic intracranial hypertension (IIH), headache pathophysiology, intracranial pressure, cerebrovascular haemodynamics, intracranial compliance

## Abstract

Calcitonin gene-related peptide (CGRP) is a key mediator in migraine pathophysiology. Idiopathic intracranial hypertension (IIH) headache phenotype is now understood to be typically migraine-like, but it is unclear whether CGRP directly provokes IIH headaches or alters intracranial pressure (ICP) dynamics.

We conducted a randomized, double-blind, placebo-controlled, two-way crossover trial to address this. Twenty women with IIH and no prior migraine were randomly assigned to receive a 20-min continuous intravenous infusion of CGRP (1.5 μg/min) or placebo (isotonic saline). The primary outcome was the difference in the proportion of participants who developed a provoked headache attack between CGRP and placebo during the 12 h observation after infusion. Secondary outcomes included the area under the curve (AUC) for headache intensity from −10 min to 12 h, the timing and duration of headache features, and baseline-adjusted changes for vital signs, cerebrovascular haemodynamics and ICP.

Seventeen participants with mean age (standard deviation) 26.7 (6.4) years completed both visits. Twelve (71%) participants developed a typical IIH headache attack with migraine-like features after CGRP compared with three (18%) after placebo (risk difference 53%; 95% confidence interval, 26–79; *P* = 0.004). The AUC_−10min-12h_ for headache intensity was higher after CGRP than after placebo (*P* = 0.016). The mean ICP remained unchanged, whereas ICP amplitude increased significantly after CGRP (*P* = 0.005). Vital signs and cerebrovascular haemodynamics AUC_−10min-90min_ were significantly altered after CGRP [increased: heart rate (*P* < 0.001), tissue oxygenation index (*P* = 0.041), and oxygenated haemoglobin (*P* < 0.001); decreased: mean arterial pressure (*P* = 0.010) and middle cerebral artery blood velocity (*P* = 0.006)].

CGRP reliably provoked typical IIH headache attacks (which have migraine-like features) and increased ICP pulse amplitude, as a measure of intracranial compliance, without altering mean pressure. These findings provide mechanistic support for CGRP involvement in headache attributed to IIH and justify prospective evaluation of CGRP pathway blockade in this population.

## Teaser

Yiangou *et al.* show that calcitonin gene-related peptide (CGRP) can provoke headache attacks in people with idiopathic intracranial hypertension (IIH) and increase intracranial pressure pulse amplitude, without raising mean pressure. CGRP blockers may therefore be a promising treatment for IIH headaches.

## Introduction

Idiopathic intracranial hypertension (IIH) is characterized by sustained elevation of intracranial pressure (ICP) with papilloedema, and no identifiable secondary cause.^1-4^ In developed countries, IIH incidence and prevalence have increased markedly over the past decade, paralleling rising obesity prevalence.^5-7^ Headache is the predominant symptom, reported by more than 80% of individuals with IIH,^8-12^ and it is a major driver of disability and reduced quality of life.^13-16^ The International Classification of Headache Disorders, third edition (ICHD-3B), defines headache attributed to IIH as a secondary headache, diagnosed by the onset or worsening of headache in the context of raised ICP and a confirmed diagnosis of IIH.^17^

Clinical and epidemiological data indicate that headache in IIH most often exhibits a migraine-like phenotype.^8,13,15,16,18-21^ The traditional assumption that IIH headaches are primarily characterized by features of raised ICP has not been supported by studies that have carefully phenotyped IIH headache.^11,18,22^ For example, when compared with a control cohort with predominant migraine, patients with IIH reported similar headache characteristics, including exacerbation with bending (50% versus 44%) and worsening in the morning (20% versus 29%).^11^ In addition, a more recent prospective study assessing headache phenotypes in patients with newly diagnosed IIH compared to suspected but non-IIH patients also reported broadly comparable features including positional headache (32% versus 37%), aggravated by straining (54% versus 62%) and relief by lumbar puncture (46% versus 30%).^22^ This is akin to other secondary headaches where it is now recognized that headaches typically mimic migraine.^8,23-26^

The mechanisms that generate headache in IIH remain incompletely understood.^27^ The frequent clinical overlap with migraine features suggests shared pathways that include activation of the trigeminovascular system and central sensitization, which are established processes in primary migraine and other secondary headache disorders.^28-30^ Calcitonin gene-related peptide (CGRP) is abundant within the trigeminovascular system and CGRP signalling is a core element in migraine pathophysiology.^29,31-33^ Given that migraine attacks are self-limiting and treatable, they provide a unique opportunity to dissect underlying molecular signalling cascades *in vivo*, a human provocation model that aims to provide mechanistic insights.^34^ A series of clinical trials using a human provocation model have examined whether exogenous administration of CGRP can trigger headache.^28,34-38^ These studies consistently show that intravenous infusion of CGRP induces migraine-like headache attacks in most people with migraine and the secondary headache disorder, post-traumatic headache, supporting a causal role of CGRP in headache generation.^28,34-38^

Initial observations also suggest CGRP involvement in IIH-related headache. In a case series of individuals with IIH who presented with headache at diagnosis, monoclonal antibody blockade of the CGRP pathway was associated with prevention of recurrent raised ICP headache during relapse.^39^ In a prospective open-label study of erenumab, a monoclonal antibody targeting the CGRP receptor, clinically meaningful improvements in headache outcomes were observed in a cohort of 55 individuals with persistent post-IIH headache and a chronic migraine-like phenotype.^40^ Two studies reported elevated serum CGRP levels in IIH patients with migraine-like headache phenotypes, although a subsequent study did not confirm this finding and it is recognized that handling, storage and processing of samples is technically challenging.^41-44^

CGRP exerts potent vasodilatory effects through actions on vascular smooth muscle cells,^45^ and its haemodynamic effects have been consistently reported in human provocation studies alongside migraine-like headache in patients with migraine.^45^ These signatures provide a coherent physiological context for assessing CGRP’s impact in IIH. Beyond systemic and cerebrovascular measures, ICP dynamics may yield additional insight. The ICP pulse amplitude reflects intracranial compliance and can increase even when mean ICP is unchanged,^46-48^ which offers a plausible link between vascular signalling and symptom generation.

Despite the clinical burden of IIH-related headache,^13-15,18^ no targeted therapy has been licensed specifically for this indication. The absence of mechanism-based treatment likely reflects gaps in our understanding of the pathways that drive headache in IIH. We hypothesized that CGRP is a key driver of headaches in IIH that are often migraine-like. To test this hypothesis, we conducted a randomized, double-blind, placebo-controlled, two-way crossover trial in women with IIH and no prior migraine. Our primary aim was to determine whether CGRP provokes typical IIH headache attacks (which typically have migraine-like features) compared with placebo. Our secondary aims were to characterize the time course and features of provoked headache and to quantify baseline-adjusted changes in vital signs, cerebrovascular haemodynamics, mean ICP and ICP pulse amplitude using continuous recordings. Through this approach, we sought to connect symptoms with haemodynamic and ICP physiology and thereby delineate a plausible CGRP-mediated mechanism for headache in IIH.

## Materials and methods

### Overview

This study (IIH Provoke: ISRCTN13251508, https://www.isrctn.com/ISRCTN13251508 registered: 24/10/2022) was approved by the National Research Ethics Committee of North East—Newcastle & North Tyneside 1 (22/NE/0081). All participants provided written informed consent to participate in the study, in accordance with the Declaration of Helsinki. Through a James Lind Alliance exercise, patients and clinicians identified establishing novel therapies and optimizing knowledge of IIH headache as key priorities^49^ and further consultation with IIH patient groups and the IIH UK charity (Registration No. 1143522) supported the study design.

### Participants

Eligible patients between 18 to 60 years were recruited if they had a previous diagnosis of IIH according to the revised diagnostic criteria,^1^ headache attributed to IIH as per the ICHD-3B,^17^ and either ongoing evidence of papilloedema [active IIH, with optic nerve swelling as measured by optical coherence tomography (OCT) peripapillary retinal nerve fibre layer (RNFL) ≥109 μm in one or both eyes^50^ or after assessment by a specialist such as neuro-ophthalmologist] or IIH in ocular remission, and were able to give informed consent. Where possible, participants with a telemetric ICP monitor *in situ* were recruited. Key exclusion criteria were: previous migraine history prior to the diagnosis of IIH; non-IIH headache >1 day per month; pregnancy or trying to conceive; significant medical co-morbidity; known cardiovascular or cerebrovascular disease; medication overuse; functioning dural venous stent or CSF shunt with no programmable valve or optic nerve sheath fenestration; and currently using glucagon-like peptide-1 receptor (GLP-1R) agonist or dipeptidyl-peptidase 4 (DPP-4) inhibitor. All potential participants attended a baseline screening/enrolment visit conducted by a physician trained in neuro-ophthalmology and specialist headache medicine, during which a full medical evaluation was performed to confirm eligibility criteria. Patients were screened and assessed for eligibility by the direct clinical care team through the electronic medical records and/or through consultation from the outpatient neuro-ophthalmology clinics, neurosurgery clinics and neurosciences wards of the University Hospital Birmingham National Health Service Foundation Trust.

### Design

We enrolled participants in a randomized, double-blind, placebo-controlled, two-way crossover trial at a single centre in Birmingham, UK. Participants with IIH were recruited and randomly assigned to receive continuous intravenous infusion of 1.5 μg/min of CGRP or placebo (isotonic saline) over 20 min on two study visits separated by more than 6 days. Doses were the same as those used in published provocation studies in migraine, cluster headache and post-traumatic headache.^35,51,52^ A paper list randomization log was prepopulated by a Clinical Trials Unit senior biostatistician with prospective study participant identification numbers, sealed and concealed from the investigators until the end of the study. Block randomization (blocks of four) was used for treatment order assignment to ensure equal numbers of participants (approximately) for each arm. Assessments at the baseline screening/enrolment visit included: assessment of eligibility criteria, demographics, medical and ophthalmic history (targeted), semi-structured headache history, dispensing and explanation of daily headache diaries, vital signs, body mass index (BMI), general physical examination (targeted), urine pregnancy test, 12-lead ECG, OCT imaging (unless performed within the previous 7 days and no change in clinical circumstances), Headache Impact Test-6 (HIT-6).^53^

Participants stopped any ICP-modifying, vasoactive, diuretic, headache-preventive, or analgesic medications at least 5.5 drug half-lives prior to each provocation visit. If a participant experienced a headache exacerbation 72 h before the provocation visits, the visit was postponed. Participants arrived at the clinical research facility between 8:00 a.m. and 10:00 a.m. having had only a light breakfast or were overnight fasted. Participants abstained from intake of coffee, tea, cocoa, cola, tobacco or nicotine containing products and alcohol for 12 h before study visit and were offered breakfast prior to the assessments.

Independent clinical research facility staff were responsible for drug preparation, allocation concealment and drug administration. Participants were informed that CGRP has the potential to induce head discomfort. No details about timing, duration or characteristics or other events were mentioned to avoid bias in reporting or expectancy effects. Study visit assessments were performed in a standardized semi-recumbent position with the patient lying on a bed with one to two pillows with the head being at a 20° to 45° angle to allow for participant comfort for the duration of the monitoring. An intravenous cannula was inserted into the antecubital vein, and a time- and volume-controlled infusion pump was used to administer either CGRP or a placebo over a 20-min period. Continuous, synchronized measurements of vital signs, cerebrovascular haemodynamics and ICP were obtained throughout each provocation study visit, with recordings beginning at baseline (10 min prior to infusion) and extending up to 4 h post-infusion. Recordings were taken 10 min (±3 min) before the start of the infusion and at 10-minute intervals (±3 min) following the start of the infusion for the duration of the inpatient study visit except when not possible (e.g. a toilet break). At each measurement interval, the recordings were collected continuously for 1–2 min.

### Headache characteristics

One investigator (A.Y.) reviewed the medical and headache history in a semi-structured interview (including review of a 28-day headache diary prior to the first provocation visit) along with vital signs and a urine pregnancy test. Information obtained were compared with the baseline visit, medical records and if any discrepancies or concerns were reconciled, the assessment was reviewed with by an additional investigator (M.T, G.T, S.P.M, A.J.S). Data on headache frequency were collected both retrospectively (semi-structured interview) and prospectively (28-day headache diary). A run-in period of at least 27 days was allowed from the enrolment visit to the first provocation visit. Baseline assessments were determined at 10 min before the start of the infusion of the provocation agent (0 min). These were recorded on a headache reporting tool every 10 min up to 4 h after the infusion by the investigator. After discharge, the headache reporting tool was completed by the participant at hourly intervals up to 12 h after the start of infusion. The reporting tool consisted of recordings of participant-reported headache severity, headache features and associated symptoms, medication intake and any adverse events. Head pain severity was recorded using an 11-point numeric rating scale (NRS): 0–10, where 0 = no pain and 10 = maximum pain. The participants also reported if head pain mimicked their usual IIH headache exacerbation.

### Vital signs and cerebrovascular haemodynamics

Vital signs of heart rate and blood pressure (BP) were recorded. The heart rate was measured using a three-lead ECG (NOVA Finapres Medical Systems, Biomedical Instruments) where possible. The BP was measured beat-by-beat using finger photoplethysmography (NOVA). The NOVA uses an upper arm cuff for calibration of the reconstructed brachial artery pressure waveform performed at baseline and at regular intervals of the recordings. In the case of an unreliable ECG signal, the heart rate was calculated from the signal of the middle cerebral artery (MCA) blood velocity or the BP waveform data. Mean arterial pressure (MAP) was estimated using the diastolic and systolic BP via the commonly used formula: MAP = diastolic BP + 1/3(systolic BP − diastolic BP).^54^ Mean blood velocity in the MCA was measured using a 2-MHz transcranial Doppler ultrasound probe on the contralateral side of the intracranial pressure sensor (to allow ICP measurements), secured in place using an adjustable headset (DWL, Compumedics Ltd). Prefrontal cortical haemodynamics were measured non-invasively using near infra-red spectroscopy (NIRO-200NX, Hamamatsu Photonics KK) from probes placed on the ipsilateral side from the transcranial Doppler. The relative concentrations of oxygenated and deoxygenated haemoglobin were obtained along with the tissue oxygenation index and total haemoglobin index. Vital signs and cerebrovascular haemodynamics were acquired continuously though an analogue-to-digital converter (PowerLab ML870; ADInstruments) at 1 kHz except when not convenient (e.g. participant taking toilet or a lunch break). Data were displayed in real-time and recorded via the analogue-to-digital converter for offline analysis using commercially available software (Lab Chart v7.3.8, ADInstruments).

### Intracranial pressure

ICP measurements were obtained from participants who had previously received an ICP M.scio telemetric sensor (Aesculap-Miethke) as part of their clinical care during ventriculoperitoneal shunt insertion for fulminant IIH, between 24/09/2019 and 07/09/2023. To negate CSF flow through the shunt apparatus and replicate ICP at disease-level conditions, the shunt valve was adjusted to the highest setting to practically occlude CSF flow. To do this prior to the start of each study visit, the ProGAV valve setting was changed to 20 cm H_2_O in all participants (the gravitational unit was fixed at 25 cm H_2_O), and at the end of the visit the value was readjusted to the participant’s previous setting. ICP measurements were collected using the system’s reader unit (Miethke M.Scio, Aesculap-Miethke) set in the ‘Fast measurement’ mode for sampling at the highest rate of up to 44 measurements per second.

ICP traces were analysed using the ICPicture software to determine mean ICP and amplitude values at each time point. Only recordings with clear pulsatility were included in the analysis of the ICP amplitude. Synchronization with other physiological recordings was confirmed visually and using a time-stamped alignment. Baseline-adjusted values were used to account for potential device drift. Data quality was assured through qualitative and quantitative inspection and exclusion of artefactual readings.

### Headache attack provocation criteria

The criteria for a provoked IIH headache attack were predefined. Several factors were considered for these. Patients with IIH headache fulfil the ICHD-3B criteria and in addition the phenotype most commonly (up to 82%) resembles migraine-like headache.^18,55^ Additionally, the rationale for including migraine-like features as part of a provoked IIH headache attack is based on well-established human headache provocation models, which have demonstrated that CGRP induces headaches with migraine-like characteristics in individuals with migraine and secondary headaches such as post-traumatic headache.^28,33,35^ Notably though, experimentally provoked headaches with migraine-like features are not spontaneous (even if they are of similar phenotype to the participant’s reported spontaneous headache attacks with migraine-like features), hence the full ICHD-3B cannot be applied.^56^ Therefore, based on the above and recently published provocation studies on other secondary headache disorders^28,35^ the following criteria were used for a CGRP-induced IIH headache exacerbation over 12 h after the intravenous infusion of the provocation agent (Supplementary Fig. 1): (i) the definition of a provoked headache is one that mimics their spontaneous IIH headache attack; (ii) if their usual IIH headache attack was migraine-like, the headache needed to fulfil at least two of the following migrainous characteristics: (a) unilateral location; (b) pulsating quality; (c) moderate to severe pain severity; and (d) aggravation by or causing avoidance of routine physical activities (e.g. walking or climbing stairs); and (iii) if their usual IIH headache attack was not migraine-like, to be defined as a provoked headache, the attack would require intake of acute analgesic.

In those with mild background headache, a provoked headache attack could still have occurred as long it fulfilled the above criteria.

Biphasic headache was defined using sampling-informed criteria as an early peak in headache severity (Tmax_1_), followed by an intervening improvement of ≥2 NRS points sustained for at least 60 min, and a subsequent increase of ≥2 NRS points constituting a late peak (Tmax_2_).

### Statistical considerations and analysis

A sample size calculation was performed using a two-sided McNemar test with a type I error rate of α = 0.10 and 80% target power, assuming discordant proportions of P01 = 0.10 (headache exacerbation on the placebo day exclusively) and P10 = 0.55 (headache exacerbation on the CGRP day exclusively), informed by prior headache provocation studies and interim feasibility assessment. These assumptions were based on a systematic meta-analysis of 27 provocation studies estimating a nocebo response at 8%,^57^ earlier migraine provocation studies reporting CGRP-induced headache exacerbation rates of approximately 60%^34,58^ and a CGRP day-exclusive provocation rate of 53% in post-traumatic headache.^35^ On this basis, the study was designed to recruit 20 participants, allowing for an anticipated 12.5% drop-out rate, with at least 17 participants completing both experimental study days required for the paired crossover analysis.

Data that were normally distributed are reported as mean ± standard deviation (SD) and data that were not, are reported as median with interquartile range (IQR). Baseline recordings on the provocation day were defined as 10 min before the start of CGRP infusion.

Primary outcome was the difference in incidence of typical IIH headache exacerbations between CGRP and placebo during a 12 h observational period post-infusion. Secondary outcomes were the differences in area under the curve (AUC) for headache intensity, characterization of headache associated features including onset and duration, and difference in baseline-adjusted changes in vital signs, cerebrovascular haemodynamics and ICP.

McNemar’s test (exact binomial, two-sided) was used to detect differences in incidence of IIH headache exacerbation, headache characteristics, associated symptoms and adverse events between study visits. AUCs for headache intensity (−10 min to 12 h), vital signs, cerebrovascular haemodynamics and ICP (−10 min to 90 and 90 min to 4 h) were calculated according to the trapezium rule^59^ to obtain summary measures for comparison between visit days. Owing to the variability in baseline measurements of the vital signs, cerebrovascular measures and ICP, the AUC calculation was based on the change from baseline after the infusion, therefore was baseline-corrected. Differences in AUC were compared between the CGRP and placebo study visit using Wilcoxon signed-rank test for headache intensity and paired *t*-test for vital signs, cerebrovascular haemodynamics and ICP. Continuous variables between the study visits or changes from the baseline were compared with a paired *t*-test or a Wilcoxon signed-rank test as appropriate. Period and carryover effects between relevant baseline clinical characteristics of study days were compared using unpaired *t*-tests and Mann-Whitney tests accordingly. No adjustments for multiple comparisons were made and a *P*-value of < 0.05 was considered as significance level. Analyses were performed with SPSS Statistics (Version 29 Armonk, NY: IBM Corp) and GraphPad Prism 10.1.2 10 (GraphPad software).

## Results

### Participant characteristics

**Figure 1:**
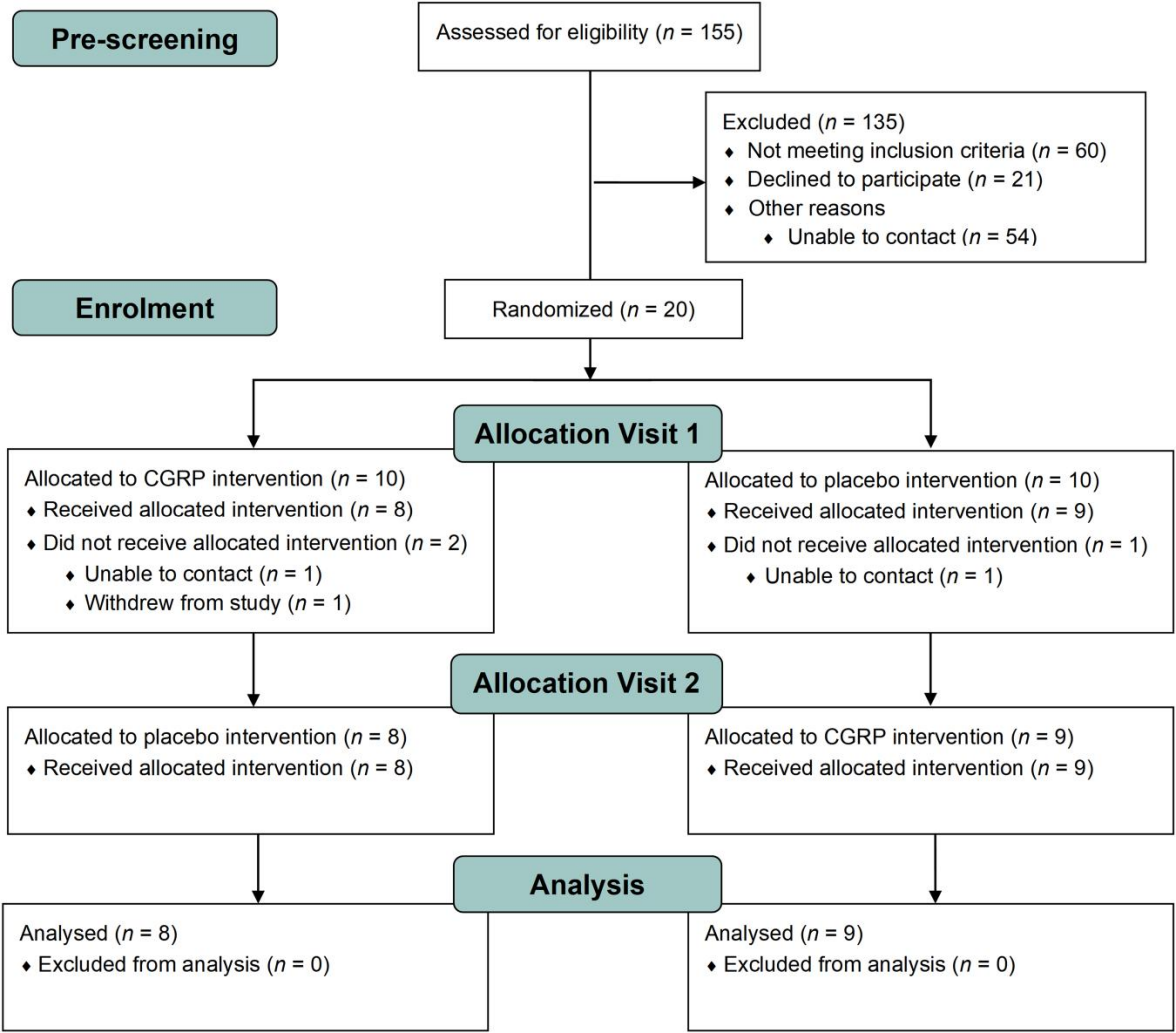
**Flow chart of the study showing participant recruitment of people with idiopathic intracranial hypertension**. CGRP = calcitonin gene-related peptide.

Twenty participants were enrolled and randomized between February 2023 to May 2024. Seventeen participants completed both provocation study days and were included in the analysis (Fig. 1). Three participants discontinued participation before receiving the study drug: one withdrew for reasons unrelated to safety and two were unable to be contacted after randomization. Baseline clinical characteristics and demographics are shown in Table 1.^50^ Mean (SD) age was 26.7 (6.4) years, weight 105.6 (23.2) kg and BMI 38.5 (8.5) kg/m^2^. Mean (SD) time from IIH diagnosis to enrolment was 20.6 (22.9) months. Most participants reported headaches with a migraine-like phenotype (*n* = 16/17, 95%). Family history of migraine was reported by four (24%) participants. The median (IQR) baseline headache severity score at 10 min before the infusion was 0 (0) for both placebo and CGRP. There were no period or carryover effects in baseline headache scores between the placebo and CGRP visits (*P* = 0.838).

**Table 1:** Demographics and clinical characteristics of the analysed study participants

Clinical characteristics	*n* = 17
Age, years	26.7 (6.4)
Weight, kg	105.6 (23.2)
BMI, kg/m^2^	38.5 (8.5)
Time from diagnosis, months	20.6 (22.9)
Papilloedema severity (OCT RNFL), μm^a^	147.1 (58.4)
Monthly headache days	24.9 (8.0)
Severity (non-migrainous headaches)	5.9 (2.8)
Monthly migraine-like days	16.2 (9.1)
Monthly migraine-like days severity (NRS)	9.1 (1.5)
Headache Impact Test 6 (HIT-6) score	58.5 (7.2)
**Demographics**	
Ethnicity	
White, British	13, 76%
Mixed, White and Black Caribbean	3, 18%
Black or Black British, African	1, 6%
Headache phenotype	
Migraine-like	16, 94%
Episodic	2, 12%
Chronic	14, 82%
Tension-type	1, 6%
Headache worse on:	
Bending forwards	13, 76%
Coughing/straining	14, 82%
Routine physical activity	14, 82%
Morning hours/lying down	14, 82%
Retrobulbar pain	14, 82%
Headache as dominating presenting feature at IIH diagnosis	16, 94%
Family history of migraine	4, 24%
Acetazolamide use	3, 18%
Migraine preventative use^b^	4, 24%
Triptan use	6, 35%
Simple analgesic use^c^	13, 76%

Values are presented as mean (standard deviation) or *n*, %. BMI = body mass index; IIH = Idiopathic intracranial hypertension; HIT-6 = Headache Impact Test-6; NRS = numeric rating scale (11-point scale, 0–10, where 0 = no pain and 10 = maximum pain); OCT = optical coherence tomography; RNFL = peripapillary retinal nerve fibre layer.

^a^OCT measured by RNFL in the worst eye at enrolment (109 μm was used as a cut off for normality and absence of papilloedema).^50^

^b^No participants had received CGRP monoclonal antibodies, CGRP antagonists or onabotulinumtoxinA.

^c^Number of participants taking opioids: four (24%).

### CGRP-induced headache exacerbation

During the 12 h recording period, intravenous infusion of CGRP provoked an IIH headache with migraine- or probable migraine-like features in 12 of 17 (71%) participants, compared to three (18%) after placebo [risk difference 53%; 95% confidence interval (CI), 26–79; *P* = 0.004] (Supplementary Table 1). These headaches mimicked their usual headache exacerbations (Supplementary Table 1). Nine of twelve participants were noted to have an IIH headache exacerbation only after CGRP and three after both CGRP and placebo. There were no participants reporting an IIH headache exacerbation solely after placebo. Six of twelve participants developed an IIH headache exacerbation within 90 min after the infusion of CGRP as opposed to none after placebo.

The four participants, that had a family history of migraine, exclusively had an IIH headache provocation after CGRP infusion and not placebo. A mild background headache at any time during the 72 h prior to the provocation visits was reported by 12 participants. Eight participants reported a mild background headache prior to both provocation visits, two participants before the CGRP visit only, and two participants prior to the placebo visit only. Eight of ten participants (80%) that had a mild background headache in the 72 h prior to the CGRP visit, experienced a headache provocation. Whilst amongst those without a headache in the 72 h prior to the CGRP visit, four from seven participants (57%) experienced a headache provocation. Among participants with active IIH (presence of papilloedema) (*n* = 9), CGRP provoked a headache attack in six participants (67%), compared with one (11%) after placebo. Among participants in ocular remission (no papilloedema) (*n* = 8), CGRP provoked an IIH headache exacerbation in six participants (75%), compared with two (25%) after placebo.

**Figure 2:**
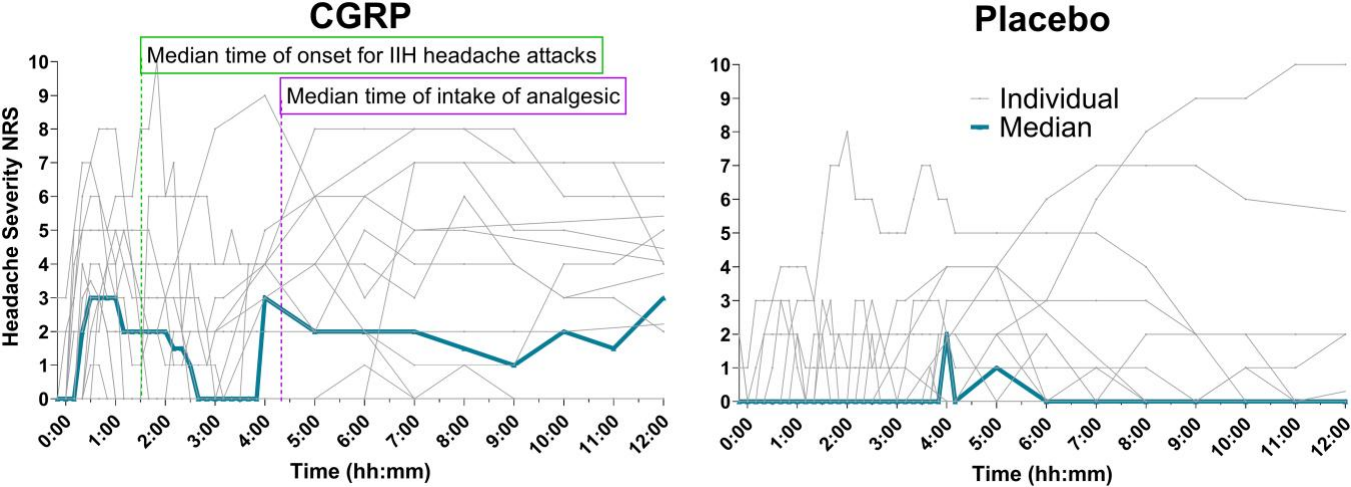
**Headache severity scores on a numeric rating scale (NRS) after calcitonin gene-related peptide (CGRP) and after placebo.** Individual severity scores on the NRS are represented by thin grey lines and the median with thick blue line.

The overall headache severity score AUC_−10min-12h_ was higher after CGRP [median (IQR): 1800 (95–2423)] compared to placebo [400 (70–952.5)] (*P* = 0.016) (Fig. 2). The median (IQR) peak severity score of any headache was higher after CGRP [6.5 (4.8–8) (*n* = 14)] compared to after placebo [3 (2–4.8) (*n* = 14)] (*P* = 0.020). The median (IQR) onset time of the provoked IIH headache exacerbation after CGRP was 90 (35–285) min (*n* = 12) whereas after placebo was 360 (100–390) min (*n* = 3) (*P* = 0.109). The median (IQR) peak severity time of the IIH headache exacerbation after CGRP was 270 (72.5–345) min (*n* = 12) and after placebo 420 (120–450) min (*n* = 3) (*P* = 0.180). The median (IQR) time of analgesic medication intake after CGRP was 260 (87.5–420) min (*n* = 9) and after placebo was 330 (315–345) min (*n* = 2) (*P* = 0.180). Nine participants demonstrated a biphasic headache course after CGRP. The median (IQR) timing of the early peak (Tmax_1_) was 30 (30–30) min, while the delayed peak (Tmax_2_) occurred at 300 (240–420) min following CGRP administration.

A higher proportion of participants reported headache with a pounding/throbbing character after CGRP, 79% (*n* = 11/14) compared to after placebo 36% (*n* = 5/14) (*P* = 0.041). The proportion of participants that reported headache with a pressure character was similar between CGRP and placebo at 93% (*n* = 13/14) and 86% (*n* = 12/14) (*P* > 0.999). Only 14% (*n* = 2/14) of participants reported headache with stabbing/sharp character after each of CGRP and placebo (*P* = 0.480).

### Associated features

**Figure 3:**
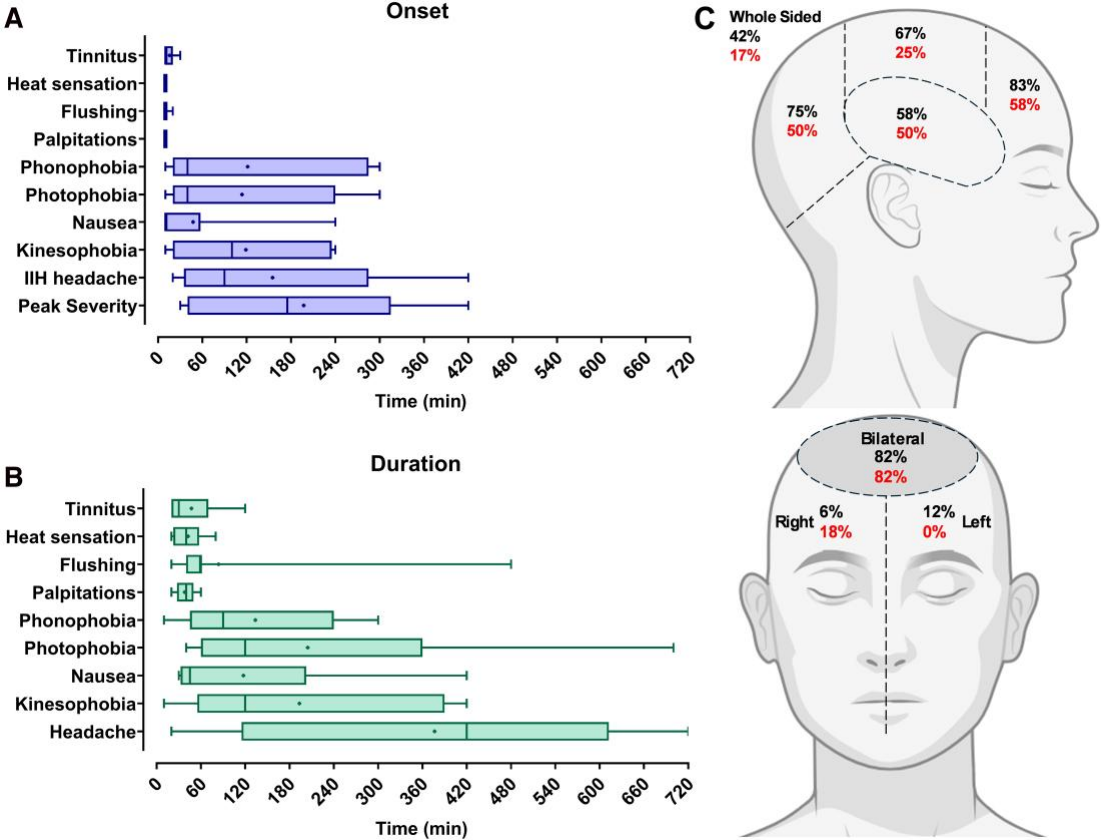
**Headache characteristics on the CGRP day.** (**A**) Plot showing the time to onset of headache features and symptoms. (**B**) Plot showing the duration of headache features and symptoms. Line inside the box represents the median, cross represents the mean, the box represents the interquartile range and the whiskers present the maximum and minimum values. (**C**) Idiopathic intracranial hypertension (IIH) headache exacerbation localization as percentage after CGRP administration. First percentages (black) represent the spontaneous IIH headache exacerbations and the second (red) represent the provoked IIH headache exacerbation after CGRP. CGRP = calcitonin gene-related peptide. Figure partially created in BioRender. Hill, L. (2026) https://BioRender.com/nui4zen.

The occurrence of specific associated features and events were different between CGRP and placebo (Table 2). The onset and duration of headache characteristics and associated features are shown in Fig. 3. The headache exacerbation after CGRP was mostly bilateral and 17% (*n* = 2/12) of participants reported headache affecting the whole side of the head.

**Table 2:** Associated features of participants after CGRP and placebo

Feature	CGRP *n*, %	Placebo *n*, %	*P*
Headache worse with activity or kinesiophobia	10, 59%	3, 18%	0.023
Nausea	8, 47%	3, 18%	0.074
Photophobia	11, 65%	4, 24%	0.023
Phonophobia	7, 41%	3, 18%	0.134
Heat sensation	16, 94%	1, 6%	<0.001
Flushing or facial redness	13, 76%	1, 6%	0.002
Palpitations	10, 59%	0	0.004
Tinnitus	6, 35%	3, 18%	0.248

### Vital signs, cerebrovascular haemodynamics and intracranial pressure

**Figure 4:**
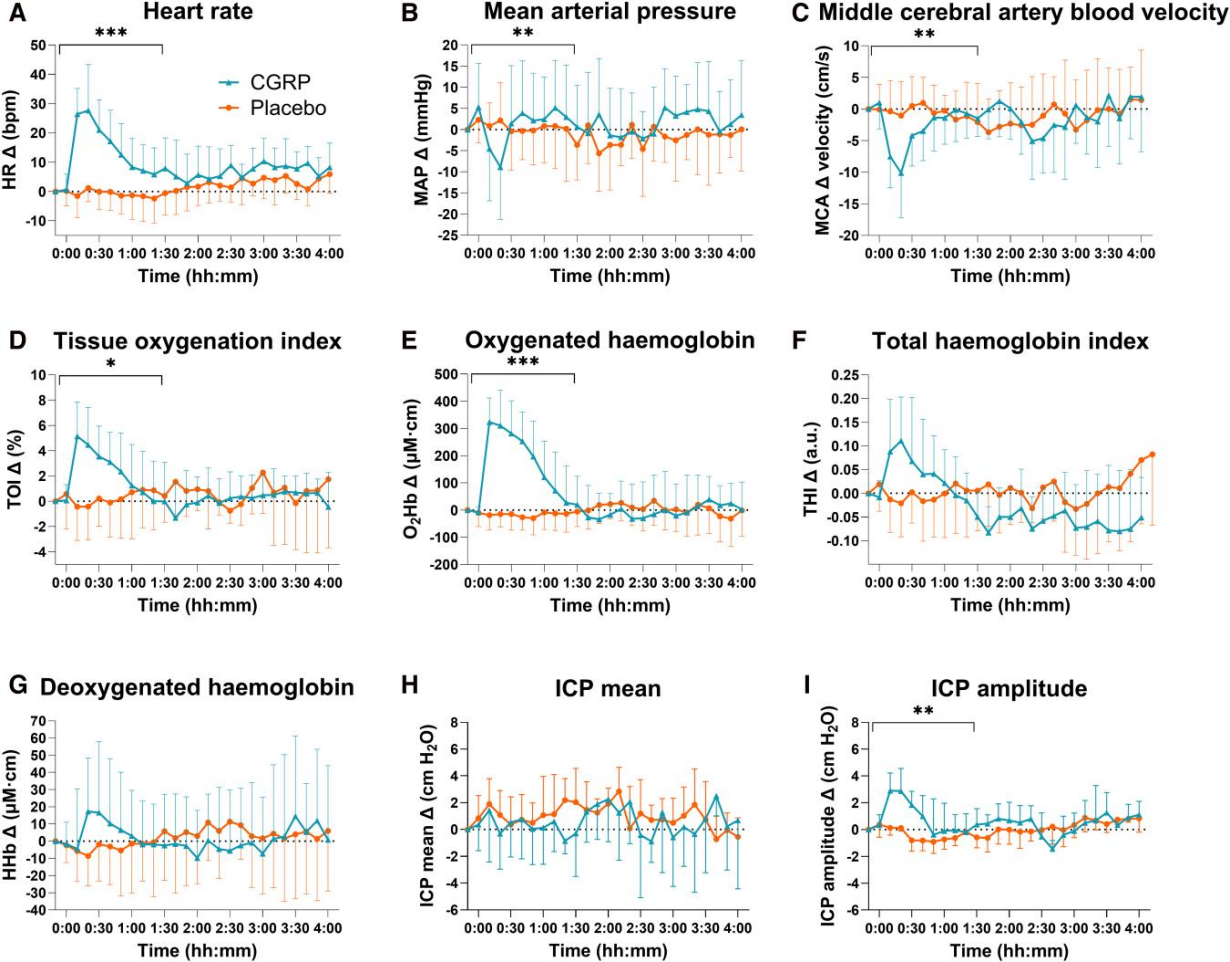
**Changes in vital signs, cerebrovascular haemodynamics and intracranial pressure after infusion of calcitonin gene-related peptide (CGRP) and placebo**. Changes from baseline after infusion of CGRP and placebo. (**A**) Heart rate (HR). (**B**) Mean arterial pressure (MAP). (**C**) Middle cerebral artery (MCA) blood velocity. (**D**) Tissue oxygenation index (TOI). (**E**) Oxygenated haemoglobin (O_2_Hb). (**F**) Total haemoglobin index (THI) (**G**) Deoxygenated haemoglobin (HHb). (**H**) Intracranial pressure (ICP) mean. (**I**) ICP amplitude. Turquoise lines with triangle points represent changes after the infusion of CGRP. Orange lines with circle points represent changes after the infusion of placebo. Data-points represent mean and error bars are standard deviation, *n* = 17 for **A**–**C**, *n* = 15 for **D**–**G**, *n* = 12 for **H** and *n* = 9 for **I**. AUC_−10min-90min_ between CGRP and placebo visits *P < 0.05, **P < 0.01, ***P < 0.001. bpm = beats per minute.

Vital signs and cerebrovascular measures revealed no period or carryover effects between the first and the second visit, and similar values between the CGRP and placebo values (Supplementary Table 2). There were significant changes noted after CGRP administration compared to placebo during the first 90 min (AUC_−10min-90min_), with no significant differences observed thereafter (AUC_90min-4h_). During the first 90 min after the infusion there was a significant increase in the heart rate, the tissue oxygenation index and oxygenated haemoglobin, and a decrease in MAP, mean MCA blood velocity after CGRP, compared to placebo (Table 3 and Fig. 4). Subanalysis of systolic and diastolic BP demonstrated changes consistent with MAP and did not alter the temporal relationship between CGRP administration and increased ICP amplitude (Supplementary Table 3 and Supplementary Fig. 2).

**Table 3:** Vital signs, cerebrovascular and intracranial pressure responses to CGRP versus placebo

Clinical variable, mean (SD), *n*	AUC_−10min-90min_			AUC_90min-4h_		
	CGRP	Placebo	*P*	CGRP	Placebo	*P*
Heart rate, beats/min	1448 (544.3), 17	435.7 (303.0), 17	<0.001	931.7 (733.7), 16	580.2 (540.9), 17	0.168
Mean arterial pressure, mmHg	836.8 (460.6), 17	493.0 (215.2), 17	0.01	916.4 (782.9), 16	658.4 (271.6), 16	0.199
Middle cerebral artery blood velocity, mean, cm/s	371.5 (210.0), 17	184.4 (193.3), 17	0.006	533.2 (362.9), 15	628.7 (391.4), 17	0.098
Total haemoglobin index, a.u.	8.649 (7.015), 15	5.316 (4.121), 15	0.144	8.014 (5.783), 15	8.028 (6.013), 15	0.994
Deoxygenated haemoglobin (HHb), μM, cm	2061 (1333), 15	1466 (1045), 15	0.206	2471 (2221), 15	2402 (2330), 15	0.933
Tissue oxygenation index, %	248.4 (156.7), 17	100.8 (185.8), 15	0.041	168.1 (144.8), 15	68.51 (33.02), 15	0.168
Oxygenated haemoglobin, μM, cm	17 853 (6128), 17	3958 (2878), 15	<0.001	8093 (7174), 15	7459 (5343), 15	0.780
ICP mean, cm, H_2_O	144.3 (110.0), 12	144.3 (93.4), 12	0.999	340.5 (165.3), 10	174.9 (90.0), 12	0.052
ICP amplitude, cm, H_2_O	140.1 (42.5), 8	60.6 (35.3), 8	0.005	136.0 (83.0), 8	68.5 (33.0), 8	0.091

Differences in the vital signs, cerebrovascular haemodynamics and intracranial pressure (ICP) area under the curve (AUC) between calcitonin gene-related peptide (CGRP) and placebo after 90 min (AUC_−10min-90min_) and from 90 min up to 4 h (AUC_90min-4h_).

Of the 17 participants, 13 had a functioning telemetric ICP monitor *in situ*, as part of the ventriculoperitoneal shunt apparatus. Seven had active IIH and six were in ocular remission. At the position adopted for the visit (semi-recumbent) the mean ICP was similar for both visits [15.1 (7.7) cm H_2_O for the placebo visits and 16.3 (7.5) cm H_2_O for the CGRP visits]. The mean (SD) value of the ICP amplitude in the semi-recumbent position was 7.9 (2.3) cm H_2_O for the placebo visit and 8.1 (1.6) cm H_2_O for the CGRP visit. There were no period or carry over effects noted in the ICP mean or the ICP amplitude between the placebo and CGRP visits (ICP mean *P* = 0.175, ICP amplitude *P* = 0.680).

There were no significant differences between the change of the mean ICP in the first 90 min (AUC_−10min-90min_) or thereafter (AUC_90min-4h_) after CGRP compared to placebo (Table 3 and Fig. 4). There was a significant difference between the change of the ICP amplitude in the first 90 min (AUC_−10min-90min_  *P* = 0.005) with no significant differences observed thereafter (AUC_90min-4h_) (Table 3 and Fig. 4). There were no pathological waveforms observed during the study visits.

## Discussion

Intravenous CGRP provocation induced typical headache attacks with migraine-like features in most participants with IIH (without prior history of migraine), demonstrating that CGRP can trigger headache attacks in this secondary disorder. CGRP infusion produced the expected systemic and cerebrovascular effects, including increased heart rate, reduced mean arterial pressure and reduced MCA blood velocity, consistent with its vasodilatory profile. In addition, novel findings included higher tissue oxygenation index and oxygenated haemoglobin levels, likely reflecting increased cortical blood flow preceding the provoked headache. Importantly, while mean ICP remained unchanged, ICP amplitude increased significantly, suggesting reduced intracranial compliance. This novel observation links vascular dynamics to symptom generation and provides a physiological basis for headache in IIH. Future clinical trials evaluating CGRP blockade as a treatment for IIH-related headache would therefore be of considerable clinical interest.

### Headache characteristics

CGRP induced IIH headache attacks in 71% (12 of 17) of participants versus 18% (3 of 17) after placebo (*P* = 0.004). These findings are comparable to previous provocation studies in migraine^34,38,58,60^ and post-traumatic headache.^28,35^ Over the 12 h observation period the AUC of the headache severity was significantly higher after CGRP compared to placebo as with other human studies in migraine and other headache disorders.^28,34,52^ The CGRP-induced headache attacks had an earlier median time of onset compared to placebo with attacks occurring at 90 min post-infusion, comparable to prior human CGRP models in post-traumatic headache.^35^ Our results also demonstrated robust within-person effect consistent with CGRP driven head pain in IIH.

A premonitory phase was not captured due to rapid headache onset (median 20 min); consistent with other CGRP infusion studies.^61^ Supporting the relevance of CGRP in driving IIH headache, it is established that, aside from the systemic vasodilatory effects, healthy controls have not been shown to experience headache attack exacerbations with migraine-like features after CGRP infusion.^51,62^ The resemblance between provoked and spontaneous IIH headaches which are predominantly migraine-like suggests that common peripheral mechanisms may underlie headache generation in both disorders. The stronger provocation among individuals with mild background pain^38^ or a family history of migraine suggests that pre-existing trigeminovascular sensitization may enhance susceptibility to CGRP-induced attacks. The biphasic pattern of provoked headache severity after CGRP, characterized by an early transient peak followed by a delayed secondary rise several hours later, mirrors delayed headache profiles described in established human provocation models^63^ and highlights the value of extended physiological monitoring. The transient decline in severity between early and late headache phases is unlikely to be attributable to analgesic use, as rescue medication was typically taken several hours (4 h) later, supporting a genuine biphasic response to CGRP rather than treatment effect.

Despite the provoked attacks frequently fulfilled migraine phenotypic criteria, this does not imply primary migraine. Headache attributed to IIH is well recognized to present with migraine-like features^8,18,64,65^ and may persist or fluctuate independently of contemporaneous ICP measurements.^66-69^ Consistent with ICHD-3,^70^ and recent large clinical analyses,^71^ these attacks are best interpreted as a secondary headache attributed to IIH with migraine-like features. This likely reflects a shared downstream trigeminovascular pathway rather than *de novo* primary migraine, with the initiating pathophysiological trigger arising from the underlying IIH disease process and raised ICP.

Although raised ICP is required for the diagnosis of IIH at presentation, ICP can fluctuate over the course of the day and hence the presence or absence of papilloedema was used to define active IIH.^18,40,72^ The comparable CGRP-provoked headache responses observed in participants with active IIH and those in ocular remission support the interpretation that CGRP-mediated mechanisms contribute to IIH-associated headache biology both during active disease and after ocular remission. This is important as significant headache morbidity continues in patients in whom papilloedema has settled.^39,40^

### Vital signs, cerebrovascular haemodynamics and intracranial pressure

We observed the well described physiological response^45^ to the CGRP infusion and noted significantly increased heart rate, a decrease in MAP and MCA blood velocity, which all reflect the vasodilatory effects of CGRP. These physiological changes were most pronounced at 20 min post-infusion prior to the peak in provoked headache severity. The heart rate increase and MAP drop was consistent with other migraine and post-traumatic headache CGRP studies.^28,35,45^ The MCA blood velocity reduction measured by transcranial doppler may partly reflect the CGRP-mediated cerebral vasodilation of the insonated MCA, but may also reflect some reduction in blood flow in response to the MAP drop.^73-76^

In this study, for the first time in a CGRP provocation model, we used near infra-red spectroscopy to evaluate prefrontal cortex perfusion and noted an increase in the tissue oxygenation index and in oxygenated haemoglobin. In the context of a lower MAP this suggests preserved cerebral autoregulation in the setting of CGRP-induced vasodilation, as it indicates an increase in cortical tissue perfusion.

This was the first study to continuously monitor ICP during an experimentally provoked headache. There were no significant changes in mean ICP following CGRP infusion reflecting intact auto-regulation and maintenance of cerebral perfusion pressure during the administration of vasoactive CGRP. Importantly, however, we observed that the CGRP infusion was associated with an increase in ICP amplitude. ICP amplitude reflects intracranial compliance and hence physiological reserve.^46-48,77^ The observed increase in ICP pulse amplitude is most likely accounted for by CGRP-induced vasodilation with alterations in cerebral arterial blood volume and reduced arterial compliance resulting in amplified waveform amplitudes.^78-80^ Therefore, the kinetic energy generated by the cardiac stroke volume at the skull base is not dissipated in the arterial wall. This is proposed to lead to a more direct transmission of the cardiac stroke volume kinetic energy or the arterial pulsations to the intracranial compartment. This may be relevant in understanding the throbbing nature of the cephalic pain observed in migraine-like headaches and may represent a mechanistic driver contributing to IIH headache.

It is interesting to note that the cerebrovascular haemodynamic and ICP changes occur in the build-up to the peak of the provoked headache emphasizing the concept of experimentally-induced physiological and behavioural uncoupling. Thus, CGRP-induced changes in physiology appear to precede the behavioural manifestation i.e. the IIH headache attack.

### Proposed mechanisms of CGRP-induced headache attack in IIH

A plausible explanation for our findings is a peripherally initiated mechanism in which CGRP-induced dilation of cranial arteries activates the trigeminovascular system. The trigeminovascular system constitutes the anatomical and physiological substrate for migraine pain and comprises meningeal arteries innervated by small-diameter sensory fibres of the ophthalmic branch of the trigeminal nerve.^60^ These afferents project centrally to the trigeminocervical complex in the brainstem, where second-order neurons relay nociceptive information to the thalamus and higher cortical areas responsible for pain perception. Activation of this system produces the characteristic topography and quality of migraine pain.^81^

CGRP is a potent dilator of cranial vessels, including meningeal and extracerebral arteries^82^ as also reflected by the haemodynamic changes and increased ICP amplitude observed in the present study. Such vascular dilation may mechanically and chemically stimulate perivascular trigeminal afferents in the dura mater, reinforcing nociceptive input to the brainstem predominantly via CGRP-mediated signalling.^82^ While co-release of additional neuropeptides such as substance P has been demonstrated in rodent models, evidence in humans remains limited and inconsistent: substance P release has been observed in induced pluripotent stem cell-derived human sensory neurons,^83^ but not consistently in analyses of native human tissues.^84^ This vascular–meningeal coupling may nevertheless provide a coherent explanation for the temporal association between CGRP infusion, altered intracranial compliance and the onset of migraine-like headache in IIH.

Notably, the temporal dissociation between the early haemodynamic effects of CGRP and the more sustained migraine-like headache suggests that vascular dilation alone may not fully account for the prolonged pain phenotype. Rather, CGRP exposure may initiate downstream processes that sustain peripheral nociceptive signalling beyond the initial vascular response. Whether CGRP directly activates nociceptors in a manner sufficient to account for headache generation remains debated. Although CGRP receptor expression has been reported in trigeminal ganglion neurons,^85^ this finding has not been consistently confirmed,^86,87^ and CGRP administration does not provoke immediate spontaneous (non-evoked) pain in humans^88,89^ or in mice.^90^ Instead, emerging evidence supports a model of sustained peripheral nociceptor sensitization,^91,92^ whereby CGRP induces delayed and prolonged increases in neuronal excitability and pain behaviours in experimental systems, potentially mediated by non-neuronal cells such as Schwann cells.^90,93^ This is consistent with growing recognition of peripheral glial contributions to pain signalling.^87,94^

In addition, evidence for a direct central action of circulating CGRP remains weak. CGRP has limited permeability across the blood–brain barrier,^95^ and none of the established human provocation agents that trigger migraine require central access to produce attacks.^81^ Moreover, the most effective migraine preventives such as CGRP-pathway antibodies^96^ and small-molecule antagonists^97^ achieve their therapeutic effect despite minimal CNS penetration, strongly supporting a peripheral site of action. Central sensitization phenomena such as cutaneous allodynia, which are frequently reported in IIH,^98^ likely represent downstream consequences of sustained peripheral activation rather than the initiating event. This interpretation is consistent with converging experimental and clinical evidence: raised ICP alters vascular pulsatility and mechanical forces in the meninges, facilitating trigeminovascular activation.^99^ Furthermore, headache severity and allodynia correlate with ICP and improve when it is reduced^55^ and blockade of the CGRP receptor alleviates headache in both experimental and clinical settings of elevated ICP.^39,99^ Collectively, these findings point to a peripheral mechanism linking vascular dynamics, meningeal afferent activation, and migraine-like headache in IIH.

Our detailed evaluation of cerebrovascular haemodynamics demonstrated for the first time increase ICP pulse amplitude with CGRP-induced provoked headaches. We hypothesized that changes in ICP amplitude, a marker of reduced intracranial compliance, could lead to amplified pulse transmission and increased activation of trigeminovascular pathways, thereby generating migraine-like pain. It is not known whether the changes in ICP amplitude are phenomenon exclusive to IIH headache as ICP has not been measured in migraine provocation studies, but it would be interesting to explore whether this is also observed in the general migraine population.

### Strengths, limitations and future directions

This study employed a rigorous randomized, double-blind, placebo-controlled, crossover design, which provided strong internal validity and permitted robust within-subject comparisons. The continuous and synchronized cerebrovascular recordings enabled a detailed and novel characterization of haemodynamic and ICP responses and provided internal consistency and mechanistic insights. The well-defined cohort along with pre-specified provocation criteria enables reproducibility and comparability with similar studies. Further, the sample size was predetermined from effect sizes reported in previous provocation studies, supporting adequate power to detect within-subject effects. Patients with prior migraine were excluded ensuring that the study exclusively included patients with only IIH headaches.

Several limitations need to be considered. Although the sample size was statistically powered for the primary outcome the ICP amplitude was only available in a subset of participants (*n* = 8) due to lack of discernible waveform data in some recordings. In addition, ICP amplitude was not defined as a primary outcome, but assessed as a supportive exploratory measure to inform mechanistic interpretation. Given the range of secondary end-points across headache outcomes, ICP and haemodynamic measures, and the absence of formal correction for multiple testing, inflation of type I error across secondary outcomes cannot be excluded including individual physiological parameters such as ICP amplitude. These exploratory findings should therefore be interpreted with caution, although the consistency of the results supports them as robust and warrants validation in future studies. Patient-reported outcome measures, such as headache diaries and reporting tools were employed to assess headache severity and associated features. Whilst these outcome measures are fundamentally subjective and may introduce bias, they are widely used in human headache model studies and align with standard clinical practice and clinical trial guidelines.^34,100^ As the participants did not have evidence of migraine prior to the IIH diagnosis, the results of this study cannot be extrapolated to women with IIH with a prior migraine diagnosis.

Future studies should evaluate CGRP blockade in headache attributed to IIH using randomized controlled trial designs with clinically meaningful end-points. Physiological measures, such as ICP amplitude and validated non-invasive ICP surrogates, may provide valuable mechanistic insights and should be considered as outcomes in future physiology studies.

## Conclusions

In this randomized, double-blind, placebo-controlled, crossover provocation study, intravenous CGRP reliably induced headache attacks in women with IIH that resembled their spontaneous headache attributed to IIH and exhibited migraine-like features. CGRP infusion was associated with increases in ICP pulse amplitude without changes in mean ICP and with higher cortical oxygenation indices, allowing temporal alignment of physiological changes and headache evolution. Taken together, these findings provide controlled experimental evidence that CGRP can provoke headache attacks in IIH through peripheral trigeminovascular mechanisms and suggest that sustained headache expression may reflect sensitization within this system rather than vascular effects alone. These observations offer mechanistic insight into shared pathways between headache attributed to IIH and migraine. Headache in IIH represents the predominant disabler and effective treatments are an unmet need. The present findings support further evaluation of CGRP-pathway inhibitors in IIH in appropriately designed randomized controlled trials.

## Supplementary Material

awag126_Supplementary_Data

## Data Availability

Data will be made available upon reasonable request from 12 months to 3 years after publication of this article, subject to approval by the original study investigators. Proposals should be directed to the corresponding author, and requesters will be required to sign a data access agreement.
