# Supplementary material for: Calcitonin gene-related peptide induces headache attacks in people with idiopathic intracranial hypertension
Source: Brain. 2026 Apr 16;149(8):2629–42. doi: 10.1093/brain/awag126 (PMC13431804; doi:10.1093/brain/awag126)
Supplement: awag126_Supplementary_Data [file awag126_supplementary_data.pdf]

Supplementary material

Supplementary material is available at *Brain* online

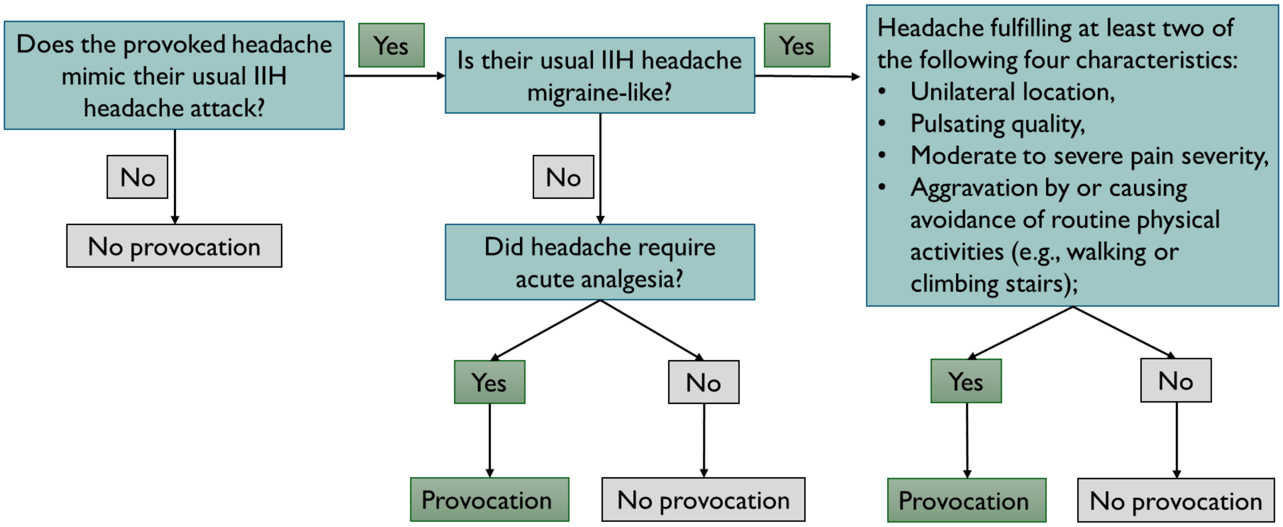

Supplementary Figure 1 Flowchart showing the criteria that were used to define a calcitonin gene-related peptide (CGRP)-induced idiopathic intracranial hypertension (IIH) headache attack.

Supplementary Table 1 Headache characteristics and associated symptoms in IIH patients after CGRP and placebo infusion over the 12 h recording period.

Patient	Occasion	Peak Headache (Duration)	Headache Characteristics[a]	Associated symptoms[b]	Mimics usual IIH headache[c], onset	ML, onset	PML, onset	Treatment (time), Efficacy
1*	Spont.	(6h)	Bl, 8, Press & Pound, +	+ / + +		Yes		
	CGRP	5h (2h)	Rt, 4, Pound, +	- / + +	Yes, 5h	Yes, 5h		
	Placebo	8h (20m)	Lt, 2, Press, -	- / - -				
3*	Spont.	(32h)	Lt, 10, Press & Pound, +	+ / + +		Yes		
	CGRP	4h (8h)	Bl, 9, Press & Pound, +	+ / + +	Yes, 30m	Yes, 30m		IB 400mg (7h), No
	Placebo	4h (2h)	Bl, 4, Press & Pound, -	- / + -				
4	Spont.	(24h)	Bl, 10, Press, +	+ / + +		Yes		
	CGRP	30m (20m)	Bl, 1, Press, -	- / - -				
	Placebo	50m (30m)	Rt, 3, Press , -	- / - -				
5	Spont.	(48h)	Bl, 10, Press & Pound, +	+ / + +		Yes		
	CGRP	30m (12h)	Bl, 7, Press & Pound, +	+ / + +	Yes, 30m	Yes, 30m		
	Placebo	3h (10h)	Lt, 8, Pound, +	+ / + +	Yes, 2h	Yes, 2h		
6*	Spont.	(72h)	Lt, 10, Press & Pound, +	+ / + +		Yes		
	CGRP	2h (2h)	Bl, 10, Press & Pound, +	+ / + +	Yes, 1h	Yes, 1h		SU 50mg, PA 1g, ME 10mg (2h), No CC 60mg/1g (4h, 10h), No
	Placebo	8h (5h)	Bl, 10, Press & Pound, -	- / + +	Yes, 7h	Yes, 7h		CC 60mg/1g (6h & 10h), No
7*	Spont	(10h)	Bl, 10, Press & Pound, +	- / + +		Yes		
	CGRP	2h (10h)	Bl, 6, Stab & Press & Pound, +	- / - -	Yes, 2h		Yes, 2h	TR 50mg (10h)(pelvic pain), No
	Placebo	4h (3h)	Bl, 2, Stab & Pound, -	- / - -				
8	Spont.	(24h)	Bl, 9, Press & Pound, +	+ / + +		Yes		
	CGRP	7h (5h)	Bl, 7, Press & Pound, -	- / - -	Yes, 7h		Yes, 7h	PA 1g (7h), No & IB 400mg (12h) Yes
	Placebo	4h (7h)	Bl, 3, Press, -	- / - -				
10*	Spont.	(24h)	Bl, 10, Press & Pound, +	- / + +		Yes		
	CGRP	None						
	Placebo	1h (10m)	Bl, 2, Press, -	- / - -				
12	Spont.	(24h)	Bl, 8, Press & Pound, +	+ / + +		Yes		
	CGRP	6h (9h)	Bl, 5, Press & Pound, +	- / + -	Yes, 4h		Yes, 4h	SU 50mg, PA 1g, ME 10mg (4h), No
	Placebo	2h (4h)	Bl, 3, Press, -	- / - -				
13*	Spont.	(24h)	Bl, 9, Press & Pound, +	+ / + +		Yes		
	CGRP	1h (1h)	Rt, 3, Press, -	+ / + -				
	Placebo	4h (1h)	Bl, 2, Press, -	- / - -				
14	Spont.	(72h)	Bl, 10, Press & Pound, +	- / + +		Yes		
	CGRP	1h (2h)	Bl, 5, Press & Pound, -	+ / + +	Yes (1h)	Yes, 1h		SU 50mg, PA 1g, OND 4mg (1h), Yes
	Placebo	5h (1h)	Lt, 4, Press, -	- / - -				PA 1g, IB 400mg (5h), Yes
15*	Spont.	(24h)	Bl, 9, Press & Pound, +	+ / + +		Yes		
	CGRP	40m (12h)	Bl, 8, Press & Pound, +	+ / + -	Yes (20m)	Yes, 40m		SU 50mg, PA 1g, OND 4mg (1h), Yes
	Placebo	6h (1h)	Bl, 2, Press, +	- / - -				
11*	Spont.	(2h)	Rt, 2, Press, -	- / - -				
	CGRP	None						
	Placebo	None						
16	Spont.	(8h)	Bl, 10, Press, +	+ / + +		Yes		
	CGRP	7h (7h)	Bl, 7, Press & Pound, +	+ / + +	Yes, 5h	Yes, 5h		IB 400mg (6h), No
	Placebo	7h (6h)	Bl, 7, Stab & Press & Pound, +	+ / + +	Yes, 6h	Yes, 6h		
18*	Spont.	(12h)	Bl, 4, Press, +	+ / - -		Yes		
	CGRP	5h (7h)	Bl, 6, Press, +	+ / + +	Yes, 4h	Yes, 4h		
	Placebo	3h (12h)	Bl, 3, Press, -	+ / - -				
19	Spont.	(24h)	Bl, 10, Press & Pound, +	+ / + +		Yes		
	CGRP	None						
	Placebo	None						
20	Spont.	(48h)	Bl, 8, Press & Pound, +	+ / + +		Yes		
	CGRP	5h (11h)	Bl, 8, Press & Pound, +	+ / + -	Yes, 1h	Yes, 1h		SU 50mg, PA 1g (3h), No RIZ 20mg (8h), No
	Placebo	None						

[a]Location/Intensity/ Character/Worse with activity or kinesiophobia
[b]Nausea/photophobia/phonophobia.
[c]Mimics IIH headache exacerbation or require acute analgesic.
Spont.: Spontaneous headache attack. Information obtained from a combination of a headache diary and semi-structured medical history interview. IIH: Idiopathic intracranial hypertension, CGRP: Calcitonin gene related peptide, ML: migraine-like, PML: probable migraine-like. Bl: Bilateral, Rt: Right, Lt: Left. PA: Paracetamol, IB: Ibuprofen, ME: Metoclopramide, SU: Sumatriptan, TR: Tramadol, CC: Co-codamol, OND: Ondansetron, RIZ: Rizatriptan, * Participants had active IIH at enrolment

Supplementary Table 2 Baseline vital signs, cerebrovascular haemodynamics and intracranial pressure at the study visits

**Cerebrovascular measures mean (SD), n**	**CGRP**	**Placebo**	**p**
Heart rate (beats/min)	76 (13), 17	76 (10), 17	0.967
Mean arterial pressure (MAP) (mmHg)	89.7 (10.1), 17	92.7 (9.4), 17	0.340
Systolic blood pressure (SBP) (mmHg)	125.9 (12.6) 17	132.7 (12.5) 17	0.085
Diastolic blood pressure (DBP) (mmHg)	71.6 (10.1) 17	72.7 (8.5) 17	0.712
Middle cerebral artery blood velocity, mean ($MCAv_{mean}$) (cm/s)	76.2 (9.5), 17	75.0 (12.5), 17	0.559
Tissue oxygenation index (TOI) (%)	68.3 (3.4), 15	69.1 (6.3), 15	0.683
Oxygenated haemoglobin (O_2Hb) µM·cm	-6.7 (74.4), 15	13.6 (46.4), 15	0.307
Total haemoglobin index (THI) (a.u.)	1.02 (0.11), 15	0.99 (0.08), 15	0.212
Deoxygenated haemoglobin (HHb) µM·cm	-2.7 (33.7), 15	-17.7 (27.3), 15	0.149
Intracranial pressure mean (cm H_2O)	16.3 (7.5), 12	15.1 (7.7), 12	0.175
Intracranial pressure amplitude (cm H_2O)	8.1 (1.6), 8	7.9 (2.3), 9	0.680

P values represent the difference between the measures at the calcitonin gene-related peptide (CGRP) and placebo visit. The near infra-red spectroscopy equipment was unavailable for two participants.

Supplementary Table 3 Differences in systolic and diastolic blood pressure area under the curve (AUC) between calcitonin gene-related peptide and placebo after 90 min ($AUC_{-10min-90min}$) and from 90min up to 4 h ($AUC_{90min-4h}$).

**Clinical Variable mean (SD), n**	**$AUC_{-10mins-90mins}$**			**$AUC_{90mins-4h}$**		
	**CGRP**	**Placebo**	**p**	**CGRP**	**Placebo**	**p**
**Systolic blood pressure (SBP) (mmHg)**	1145 (641.3), 17	644.8 (318.6), 17	**0.007***	1048 (953.4), 16	1039 (544.1), 16	0.972
**Diastolic blood pressure (SBP) (mmHg)**	842.7 (422.2), 17	518.1 (203.5), 17	**0.005***	835.4 (692.8), 16	527.8 (300.5), 16	0.122

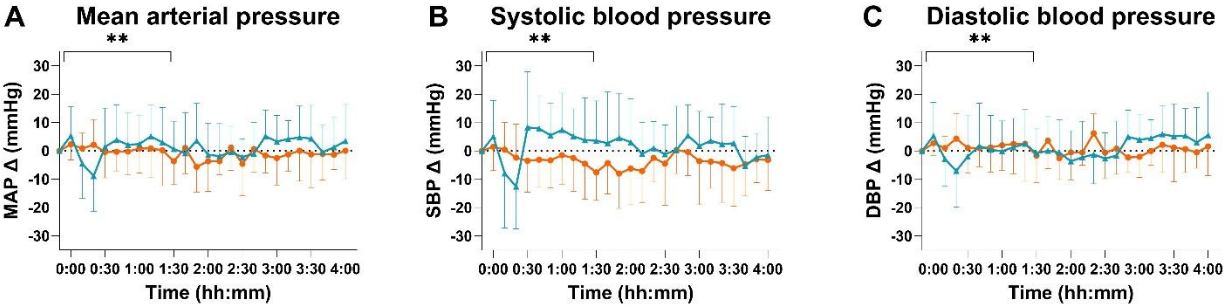

Supplementary Figure 2 Changes in blood pressure after infusion of calcitonin gene-related peptide and placebo. A. Mean arterial pressure (MAP). B. Systolic blood pressure (SBP) C. Diastolic blood pressure (DBP). D. Turquoise lines with triangle points represent changes after the infusion of CGRP. Orange lines with circle points represent changes after the infusion of placebo. Data points represent mean and error bars are SD, n = 17. $AUC_{-10min-90min}$ between CGRP and placebo visit * $P < 0.05$, ** $P < 0.01$, *** $P < 0.001$.
